# The impact of multistrain probiotics (Leuconostoc mesenteroides, Lactococcus lactis) on African catfish (Clarias gariepinus) gut microbiota, immunological response, and growth performance

**DOI:** 10.3389/fimmu.2025.1625199

**Published:** 2025-08-27

**Authors:** Assel Paritova, Altay Ussenbayev, Rauan Abdessan, Zhenisgul Seitkaliyevna Assauova, Gulnur Berikovna Kuzembekova, Gulzhan Omarbekova, Aiken Sansyzbaevna Karabassova, Aigul Jenisbekkyzy Kassen, Zhuldyzay Kenzhebekova, Gulmira Alibayevna Abulgazimova, Aruzhan Saingaliyeva

**Affiliations:** ^1^ Department of Veterinary Sanitation, S.Seifullin Kazakh AgroTechnical Research University, Astana, Kazakhstan; ^2^ Department of Veterinary Medicine, S.Seifullin Kazakh AgroTechnical Research University, Astana, Kazakhstan; ^3^ Northwest A&F University, College of Animal Science and Technology, Yangling, China; ^4^ Kazakh National Research Agrarian University, Almaty, Kazakhstan; ^5^ Virology Laboratory, Kazakh Research Institute of Veterinary Science, Almaty, Kazakhstan

**Keywords:** African clarify catfish, Leuconostoc mesenteroides, Lactococcus lactis, probiotic, immunity, growth

## Abstract

**Introduction:**

Aquaculture is globally recognized as an effective means of supporting economic growth and livelihood security. However, the sector continues to fall short of projected production targets due to the use of low-quality inputs and inadequate culture techniques. The use of probiotics in aquaculture has been proposed as a nutritional strategy to enhance fish growth and immune responses. This study investigated the effects of dietary supplementation with a multistrain probiotic combination—Leuconostoc mesenteroides and Lactococcus lactis—on growth performance, immune parameters, and gut microbiota in African catfish (Clarias gariepinus).

**Methods:**

Fish were fed either a basal diet or an experimental diet containing L. mesenteroides a 95 nd L. lactis at 10^6^ CFU/g for 8 weeks.

**Results and discussion:**

The probiotic-supplemented group showed significant improvements in feed utilization, alternative complement pathway activity, lactic acid bacterial populations, mucus secretion, and peroxidase activity compared with the control group (p < 0.05). Serum lysozyme activity was also significantly higher in the probiotic-fed group. Furthermore, fish receiving the supplemented diet exhibited superior growth metrics, including weight gain, final body weight, and specific growth rate (p < 0.05). Enhanced superoxide dismutase activity was also observed in the probiotic group. These results suggest that dietary inclusion of L. mesenteroides and L. lactis may serve as an effective immunostimulant feed additive for African catfish aquaculture.

## Introduction

Over the past two decades, aquaculture has expanded significantly and now plays a crucial role in meeting global protein demands. However, the intensification of aquaculture systems has introduced challenges related to fish health, including growth disorders, increased mortality, and disease outbreaks (1–3).

In response to these issues, antibiotics have often been used to control infections and improve productivity (4). Nevertheless, the widespread use of antibiotics poses environmental and biological risks, including the development of antimicrobial-resistant bacteria and disruption of the intestinal microbiota in aquatic organisms (5).

To address these concerns, recent studies have focused on dietary alternatives such as probiotics, which are defined as live microorganisms that, when administered in adequate amounts, confer health benefits to the host (6–10). In aquaculture, probiotics have been shown to increase nutrient absorption, stimulate immune responses, and modulate the gut microbiota of fish (11–15). These benefits are achieved through multiple mechanisms, including enzyme secretion, microbial competition, and improved intestinal integrity (16).

Probiotics can be delivered through various routes, including incorporation into feed, direct addition to water, or injection (17–21). Research has shown that specific strains, including Leuconostoc mesenteroides and Lactococcus lactis, have immunostimulatory effects when administered to Nile tilapia (Oreochromis niloticus) (2).

On the basis of these findings, the present study investigated the effects of dietary supplementation with L. mesenteroides and L. lactis on the growth performance, innate immunity, and gut microbial composition of African catfish (Clarias gariepinus). These lactic acid bacteria were originally isolated from the intestinal tracts of freshwater fish and were selected based on their probiotic potential.

This study evaluated the efficacy of multistrain probiotic supplementation as a sustainable, health-promoting feed additive in freshwater aquaculture systems.

## Materials and methods

### Animal ethics

All experimental procedures were conducted in accordance with the European Convention for the Protection of Vertebrate Animals Used for Experimental and Other Scientific Purposes (14). The protocol was approved by the Ethics Committee of Seifullin Kazakh Agrotechnical University (permit number: 2).

### Experimental design

A total of 256 healthy juvenile African catfish (C. gariepinus) with an average weight of 498 ± 2 g were obtained from a local commercial hatchery. The fish were randomly assigned to control and treatment groups (128 fish per group) and distributed into six 100 L tanks (32 fish per tank), with 2 replicate tanks per group. All tanks were equipped with a continuous flow-through freshwater system, aeration, and appropriate inlet and outlet systems. Prior to the experiment, the fish were acclimated for 14 days in a 500 L tank with aerated water (dissolved oxygen: 5 mg/L). Water quality was monitored throughout the trial. The temperature was maintained at 27°C ± 0.5°C using a digital thermometer, and pH was measured using a portable pH meter (Fisherbrand AP115). The fish were fed a control diet (without probiotics) twice daily at 09:00 and 17:00 during the acclimation period. After the 40-day feeding trial, fish were fasted for 24 hours prior to sampling. Growth performance, survival, immune parameters, and the composition of the microbiota were assessed.

### Isolation of probiotics

L. mesenteroides and L. lactis strains were isolated from the intestinal contents of common carp (Cyprinus carpio) fingerlings, as previously described (2). Briefly, 50 μL of intestinal content was diluted in distilled water at a 1:10 ratio, and 100 μL of the dilution was plated onto MRS agar using sterile pipettes. The plates were incubated at 37°C for 48 hours. Uniform colony distribution was achieved using sterilized glass beads. The colonies were further purified, and the isolates were confirmed by PCR.

### PCR confirmation

Genomic DNA was extracted using the GeneJET PCR Purification Kit (Thermo Fisher Scientific, USA), following the manufacturer’s instructions. The reference strains L. mesenteroides (NCTC AB023246) and L. lactis (NCTC M58837) were used as positive controls. Multiplex PCR (mPCR) targeting the 16S rRNA gene was performed as described by Ali and Kot (15) using an Eppendorf Mastercycler. The amplification parameters were as follows: initial denaturation at 95°C for 2.5 min; 35 cycles of 95°C for 1 min, 55°C for 1 min, and 72°C for 1 min 20 sec; and a final extension at 72°C for 2 min. PCR products were analyzed by 1% agarose gel electrophoresis stained with ethidium bromide.

### Preparation of experimental diets

The basal diet consisted of fishmeal, soybean meal, blood meal, milled wheat, extruded peas, potato starch, fish oil, premix, yeast, and gelatin (**Table 1**). The experimental diet was prepared by adding 1 × 10^7^ CFU/g of both L. mesenteroides and L. lactis via surface spraying. A 165 g portion of gelatin was dissolved in 500 mL of distilled water and applied to the diet pellets to ensure bacterial adhesion. The feed was air-dried overnight and stored at −4°C until use.

**Table 1:** Formulation of the basal diet for African catfish, Clarias gariepinus.

Feed ingredients	g kg^-1^	%
Fishmeal	30	3
Soybean meal	30	3
Blood meal	10	1
Milled wheat	7	0.7
Extruded peas	8	0.8
Potato starch	6.7	0.67
Fish oil	4	0.4
Vitamin/Mineral mixture	1.1	0.11
Premix	2.0	0.2
Yeast	0.2	0.02
Gelatin	1	0.1
Proximate composition %		
Moisture	12.1	
Crude protein	44.2	
Crude lipid	8.20	
Crude ash	7.54	

### Growth performance

Weight gain (WG), specific growth rate (SGR), feed conversion ratio (FCR), and protein efficiency ratio (PER) were calculated as described by Allameh and Yusoff (19). The following formulas were used:


$$WG\;\left(g\right)\;=\;Final\;weight\;-\;Initial\;weight$$

$$\text{SGR}\;(\%)\;=100\times \;(\ln\;{\text{W}}_{2}-\ln\;{\text{W}}_{1})/\text{T}$$

FCR = Feed intake/Weight gain

PER = Weight gain/protein intake

where W_1_ and W_2_ are the initial and final weights, respectively, and T is the number of feeding days.

### Determination of intestinal bacteria activities

Thirteen fish per group were randomly selected and euthanized after a 24-hour fasting period. Euthanasia was performed in accordance with the Animal Care and Use Committee (ACUC) Guidelines and the Standard Operating Procedure for MS-222 (tricaine methanesulfonate) for euthanasia. Fish were euthanized via anesthetic overdose (MS-222 at 250 mg/L) followed by decapitation. To ensure complete euthanasia, fish were placed in the MS-222 buffer solution for at least 10 min after gill movement had ceased (respiratory arrest), before extraction and decapitation.

The external surfaces of the fish were sterilized with 70% ethanol. The intestinal tracts were excised aseptically, rinsed with phosphate-buffered saline (PBS), and homogenized. Serial dilutions were prepared in PBS, and 100 μL aliquots were plated onto tryptic soy agar (TSA) for total bacterial counts and MRS agar for lactic acid bacteria. Plates were incubated at 25°C for 3–5 days. Colony-forming units (CFUs) were enumerated as described by Ramos and Batista (17).

### Immune responses assays

Alternative complement pathway activity (ACH_50_) was measured following the method of Yano (20). Briefly, 0.1 mL of serum was mixed with barbitone buffer to reach a final volume of 0.25 mL. Then, 0.1 mL of rabbit red blood cells (RaRBCs) was added, and the mixture was incubated for 1 h at 20°C. After incubation, 3.15 mL of 0.9% NaCl solution was added, and the tubes were centrifuged at 1,600 × g for 10 min at 4°C. The optical density (OD) of the supernatant was measured at 414 nm using a spectrophotometer, and a lysis curve was constructed. The serum dilution resulting in 50% hemolysis was used to determine complement activity. The bactericidal activity of the serum and mucus was evaluated as described by Dawood and Koshio (12). Samples were diluted 1:1 in Tris buffer (pH 7.5) and incubated with a suspension of Escherichia coli (strain IAM1239; RIBSP, Kazakhstan) at a concentration of 0.001 mg/mL for 12 h at room temperature. Bacterial viability was assessed by calculating the number of colony-forming units (CFUs) on agar plates.

Lysozyme activity in the serum and mucus was determined via turbidimetric assay. A 10 μL aliquot of each sample was mixed with 190 μL of Micrococcus lysodeikticus suspension (0.2 mg/mL in PBS, pH 7.4) in a 96-well microplate and incubated at 24°C for 5 min. The decrease in absorbance at 450 nm was measured using a microplate reader (Biotek 800 TS, Agilent Technologies, USA). One unit of lysozyme activity was defined as a decrease in OD of 0.001 per minute.

Peroxidase activity was assessed as described by Salinas and Abelli (21). A mixture of 15 μL of serum and 35 μL of Hank’s buffered salt solution was added to a 96-well plate. Then, 50 μL of 3,3′,5,5′-tetramethylbenzidine (TMB; Thermo Scientific, USA) was added to each well. After 20 min of incubation at room temperature, the reaction was stopped by adding 50 μL of 2 M sulfuric acid. The absorbance was read at 450 nm using a microplate reader.

Superoxide dismutase (SOD) activity in the serum was determined using the Ransod assay kit (Randox, Crumlin, UK) as described by Sun and Yang (22). Enzyme activity was expressed in units per milligram of protein, where one unit was defined as the amount of enzyme required to inhibit the reduction of nitroblue tetrazolium (NBT) by 50% at 550 nm.

### Statistical analysis

Data were analyzed using Prism 9 (GraphPad Software, San Diego, CA, USA). Student’s t test and one-way ANOVA followed by Tukey’s *post hoc* test were applied. Results are expressed as means ± standard errors of the mean (SEMs). Differences were considered statistically significant at p < 0.05.

## Results

### Growth performance, nutrient utilization, and fish survival

The effects of probiotic supplementation on growth performance, nutrient utilization, and fish survival are presented in **Table 2**. Compared with the control group, fish fed a diet supplemented with L. mesenteroides and L. lactis exhibited significantly greater weight gain (WG), final body weight (FBW), and specific growth rate (SGR) (p < 0.05).

**Table 2:** Growth performance parameters of African catfish (Clarias gariepinus) fed diets supplemented with L.mesenteroides and L.lactis for 40 days.

Growth performance parameters	Diets		P-value
	Control	Mixture of Leuconostoc mesenteroides and Lactococcus lactis probiotics	
^1^IBW (g)	501.3 ± 1.23	506.2 ± 1.26	0.902
^2^FBW (g)	649 ± 2.12	821 ± 3.37^*^	0.034
^3^WG (%)	147.7 ± 0.89	314.8 ± 2.11^*^	0.042
^4^SGR (%)	3.69 ± 0.18	7.87 ± 0.2^*^	0.03
^5^FI (g)	15,6 ± 0.21	15.2 ± 0.8	0.998
^6^FER	4.2 ± 0.3	1.93 ± 0.3^*^	0.024
^7^PER	0.95 ± 0.2	1.67 ± 0.02^*^	0.037
^8^PG	156.12 ± 6.7	188.3 ± 4.13^*^	0.012

^1^IBW, initial body weight; ^2^FBW, final body weight; ^3^WG, weight gain; ^4^SGR, specific growth rate; ^5^FI, feed intake; ^6^FER, feed efficiency ratio; ^7^PER, protein efficiency ratio; ^8^PG, protein gain. P ≤ 0.05 indicates statistically significant difference. Data are presented as means ± standard error of the mean (SEM) from triplicate groups.

In addition, the protein efficiency ratio (PER) and feed efficiency ratio (FER) were significantly improved in the probiotic-fed group (p < 0.05), indicating increased nutrient utilization.

A significant increase in protein gain (PG) was also observed in fish fed the diet containing L. mesenteroides and L. lactis compared with the control group (p < 0.05). Although feed intake (FI) was slightly higher in the control group, the difference was not significant (p > 0.05). Survival remained at 100% in all experimental groups, with no mortality recorded throughout the trial.

### Microbiological analyses of the gut microbiota

**Figure 1:**
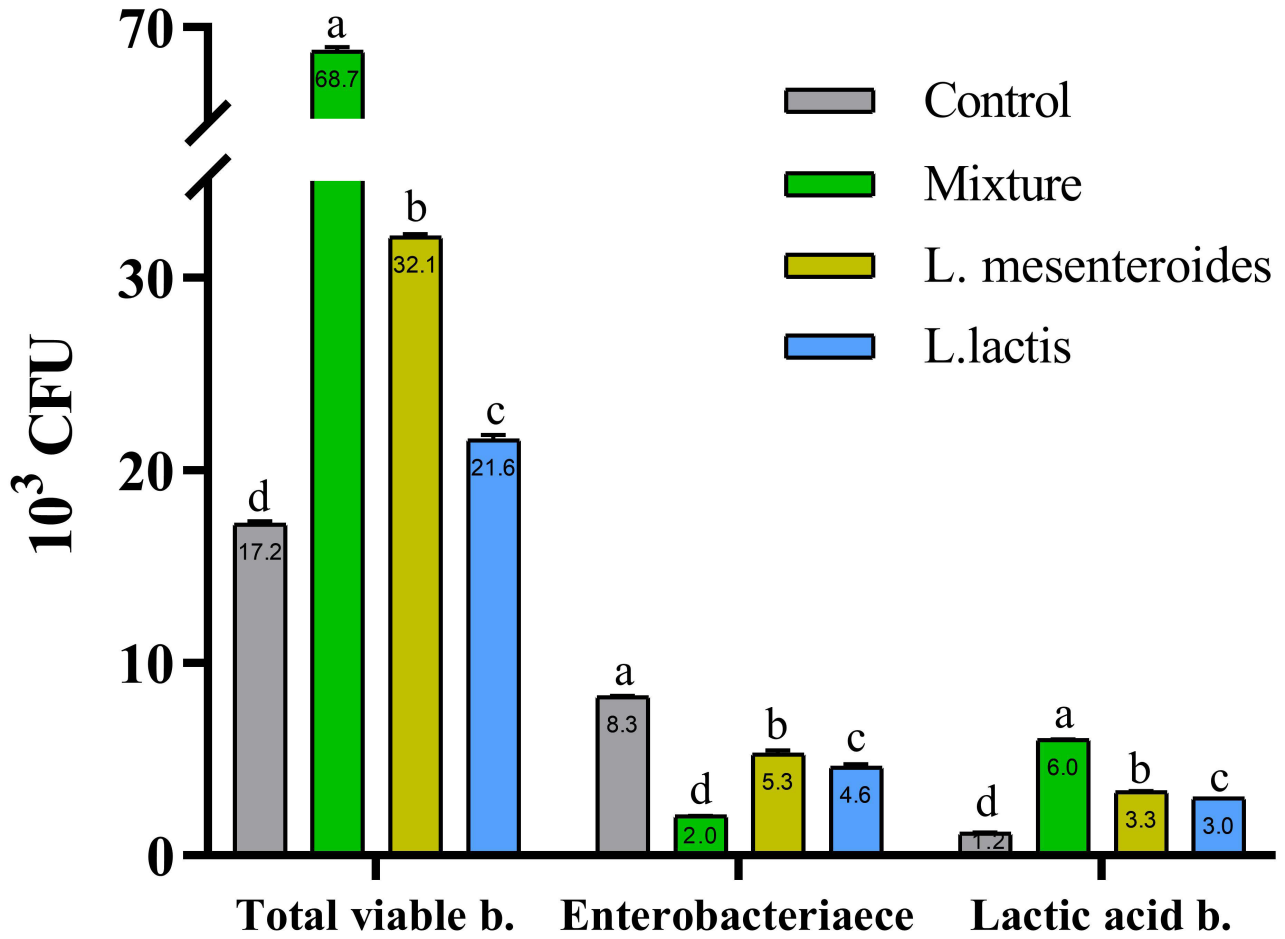
Total intestinal bacterial and lactic acid bacteria (LAB) counts in African catfish from control and probiotic-supplemented groups after 8 weeks of feeding. Data are presented as mean ± SEM from two independent experiments (n = 20 fish per group). Bars with different letters indicate statistically significant differences (p ≤ 0.05); bars sharing the same letter are not significantly different (p ≥ 0.05). L. mesenteroides – Leuconostoc mesenteroides; L. lactis – Lactococcus lactis.

Prior to the feeding trial, no detectable levels of lactic acid bacteria (LAB) were detected in the intestinal microbiota of fish in either group. However, compared with the control group, fish fed diets supplemented with L. mesenteroides and L. lactis exhibited significantly greater intestinal LAB counts (p < 0.05; **Figure 1**). In contrast, no LAB colonies were recovered from the intestines of the fish in the control group.

### Immune response development

**Figure 2:**
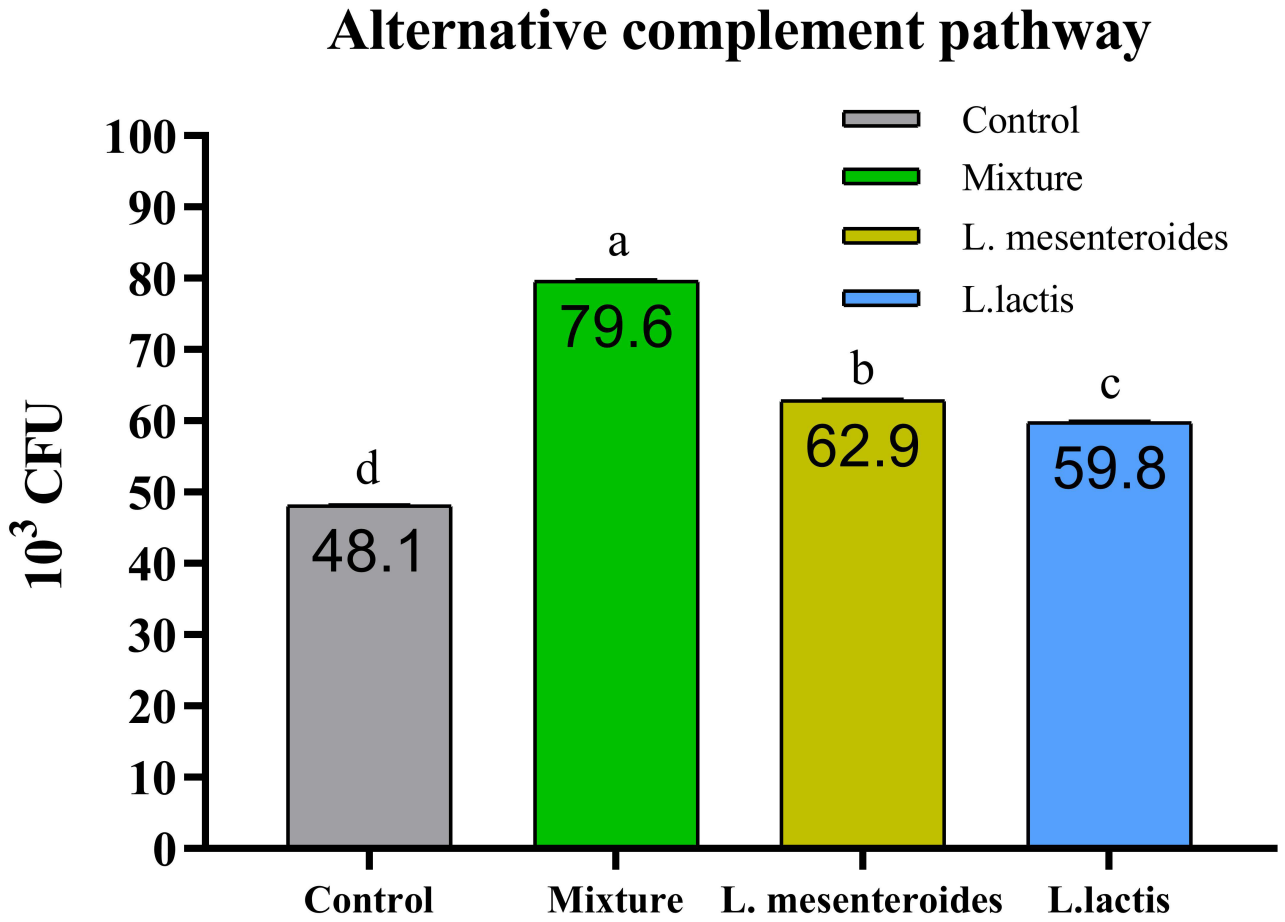
Alternative complement pathway (ACP) activity in African catfish (Clarias gariepinus) from control and probiotic-supplemented groups. Data are presented as mean ± SEM from two independent experiments (n = 20 fish per group). Bars with different letters indicate statistically significant differences (p ≤ 0.05). Bars with different letters indicate statistically significantly differences (p ≥ 0.05). L. mesenteroides – Leuconostoc mesenteroides; L. lactis – Lactococcus lactis.

**Figure 3:**
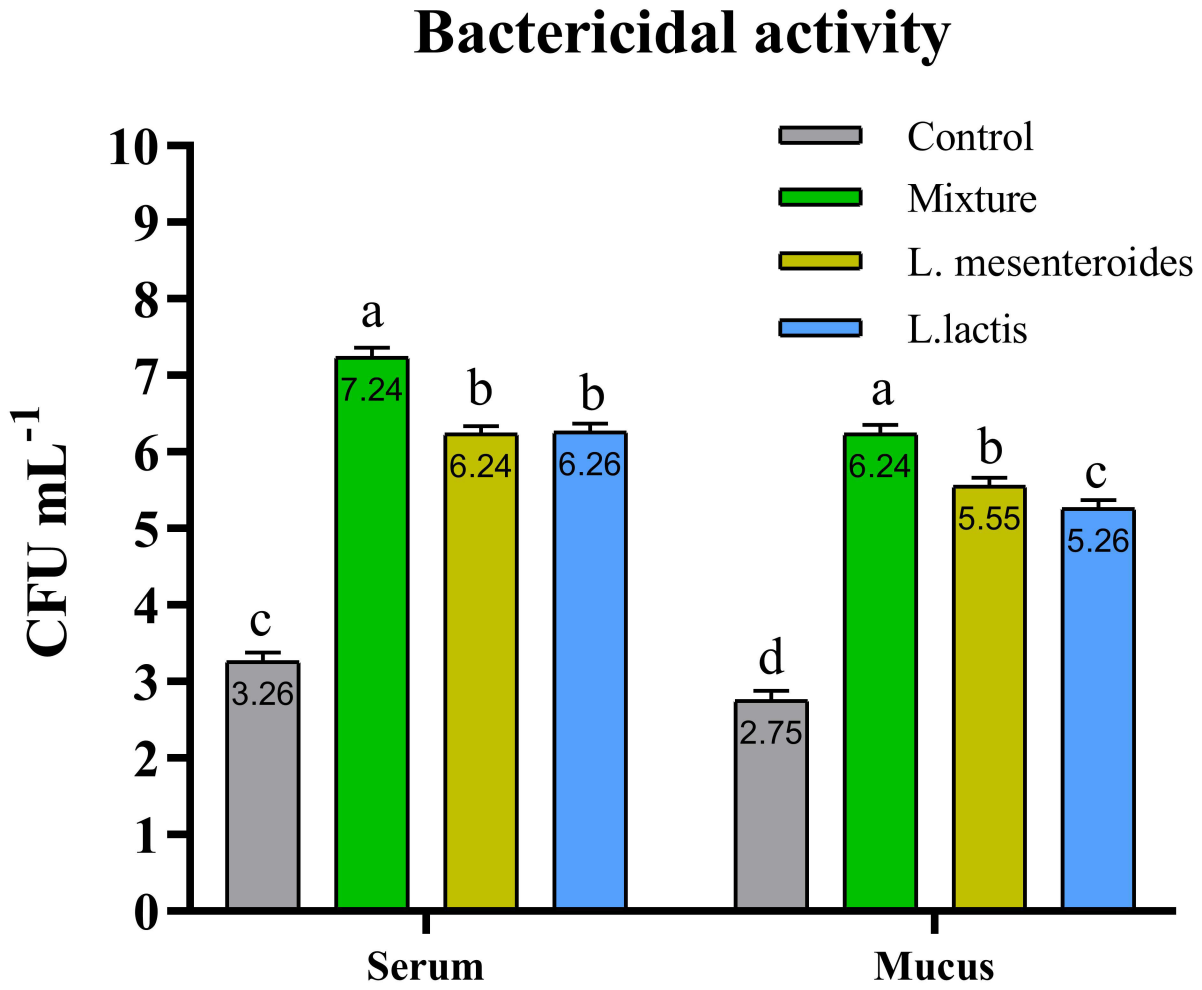
Serum and mucus bactericidal activity in African catfish (Clarias gariepinus) from control and probiotic-treated groups. Data are presented as mean ± SEM from two independent experiments (n = 20 fish per group). Bars with different letters indicate statistically significant differences (p ≤ 0.05); bars sharing the same letter are not significantly different (p ≥ 0.05). L. mesenteroides – Leuconostoc mesenteroides; L. lactis – Lactococcus lactis.

**Figure 4:**
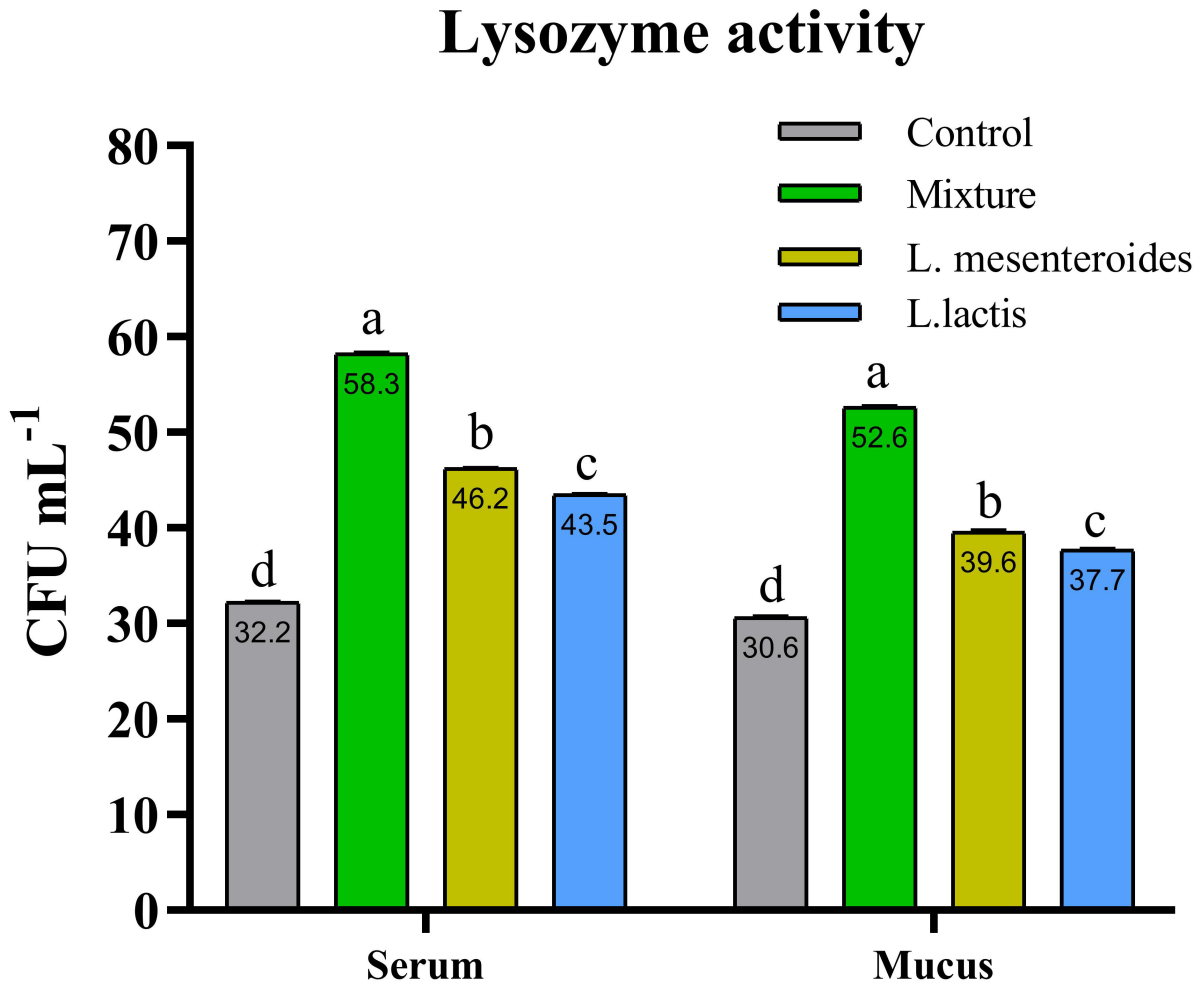
Serum and mucus lysozyme activity in African catfish (Clarias gariepinus) from control and experimental groups. Data are shown as the mean ± SEM from two independent experiments (n = 20 fish/group). Bars with different letters are variated significantly (p ≤ 0.05) and with the same alphabet have an insignificant difference (p ≥ 0.05). L.mesenteroides - Leuconostoc mesenteroides; L. lactis - Lactococcus lactis.

**Figure 5:**
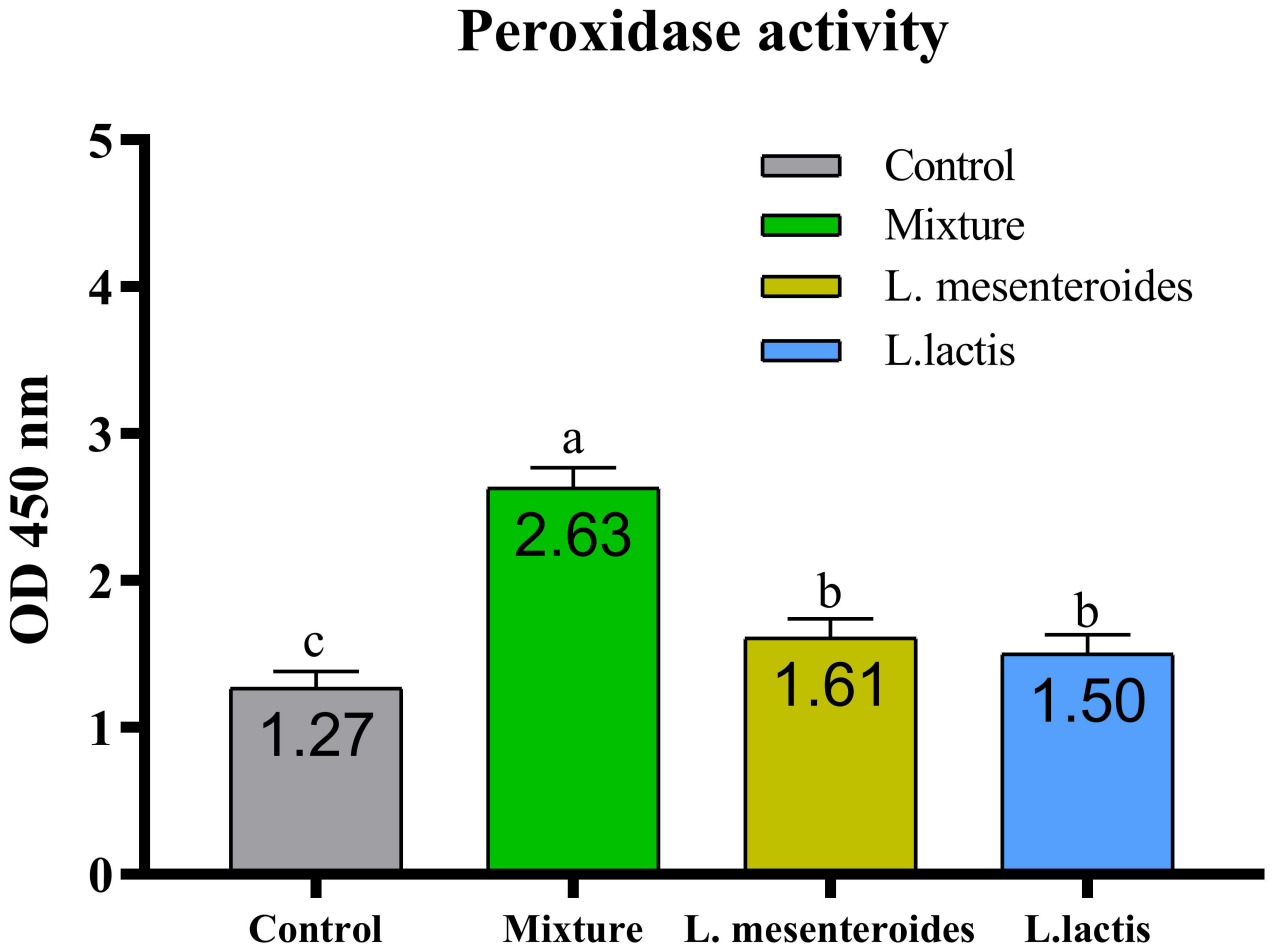
Serum peroxidase activity in African catfish (Clarias gariepinus) from control and probiotic-treated groups. Data are presented as mean ± SEM from two independent experiments (n = 20 fish per group). Bars with different letters indicate statistically significant differences (p ≤ 0.05); bars sharing the same letter are not significantly different (p ≥ 0.05). L. mesenteroides – Leuconostoc mesenteroides; L. lactis – Lactococcus lactis.

**Figure 6:**
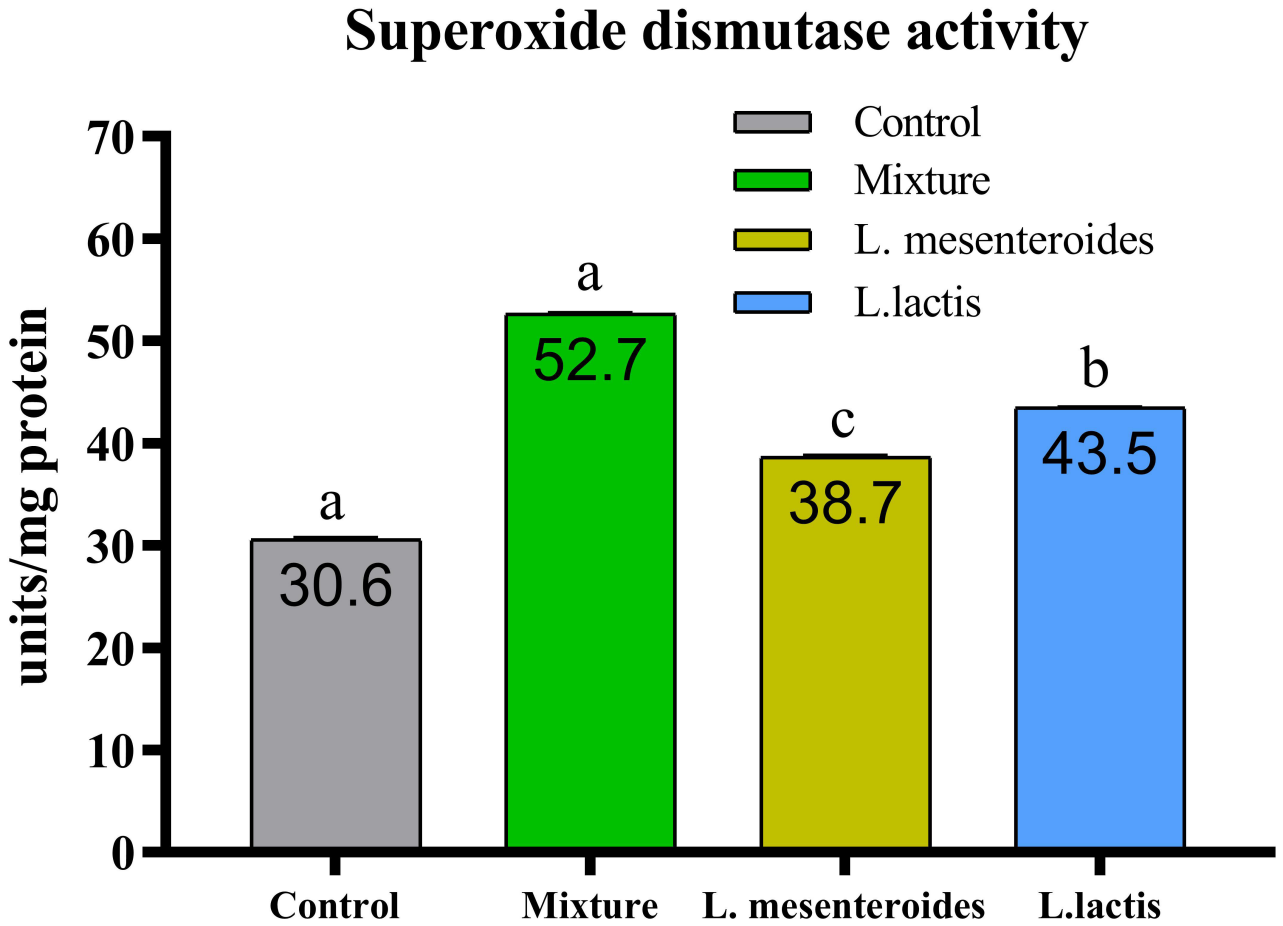
Superoxide dismutase (SOD) activity in African catfish (Clarias gariepinus) from control and probiotic-treated groups. Data are presented as mean ± SEM from two independent experiments (n = 20 fish per group). Bars with different letters indicate statistically significant differences (p ≤ 0.05); bars sharing the same letter are not significantly different (p ≥ 0.05) L.mesenteroides - Leuconostoc mesenteroides; L. lactis - Lactococcus lactis.

**Figure 7:**
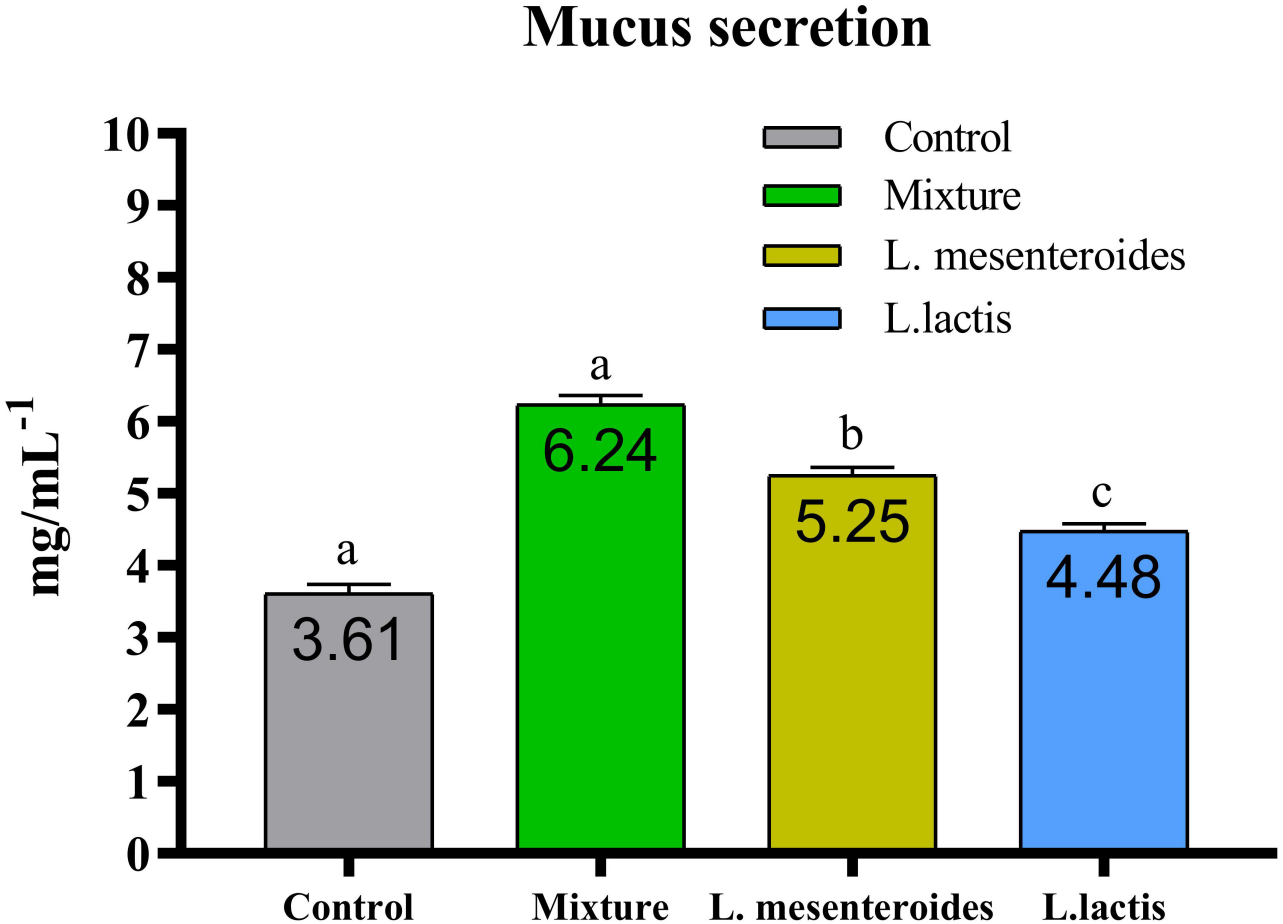
Effect of probiotic-supplemented diets on mucus secretion in African catfish (Clarias gariepinus). Data are presented as mean ± SEM from two independent experiments (n = 20 fish per group). Bars with different letters indicate statistically significant differences (p ≤ 0.05); bars sharing the same letter are not significantly different (p ≥ 0.05). L.mesenteroides - Leuconostoc mesenteroides; L. lactis - Lactococcus lactis.

Compared with the control group, alternative complement pathway (ACP) activity was lowest in the first control group of fish fed the ordinary diet. ACP activity increased in all groups of fish supplemented with probiotics. The highest ACP level was observed in the second group receiving a mixture of Lactobacillus mesenteroides and Lactobacillus lactis. ACP levels in the third and fourth groups fed with mono-probiotic diets (L. mesenteroides alone and L. lactis alone, respectively) were lower (**Figure 2**). Similarly, other innate immune parameters were significantly increased in the probiotic-supplemented groups. Serum and mucus bactericidal activity (**Figure 3**), lysozyme activity (**Figure 4**), and peroxidase activity (**Figure 5**) were all markedly elevated compared with the control group. In addition, SOD activity was significantly higher in fish fed the probiotic mixture (p ≤ 0.05; **Figure 6**). Mucus secretion was also significantly increased in this group, indicating an improvement in the mucosal immune response (p ≤ 0.05; **Figure 7**).

## Discussion

Numerous studies have investigated the application of LAB individual strains as probiotics to enhance growth performance, immune responses, and gut microbiota composition in fish species (22–26). However, comparatively less attention has been given to evaluating the synergistic effects of multistrain LAB probiotics on fish health and physiology (2, 27–30). The present study assessed the effects of dietary supplementation with L. mesenteroides and L. lactis on the growth performance, immune function, and gut microbiota of African catfish (C. gariepinus). The results demonstrated that compared with the control, the 8-week probiotic combination significantly improved both growth rates and innate immune responses.

Similarly, the use of various Lactobacillus species as dietary supplements has been shown to enhance growth performance in several fish species, including olive flounder (Paralichthys olivaceus) (27), Nile tilapia (Oreochromis spp.) (29), and gilthead sea bream (Sparus aurata) (30). Moreover, improved growth performance has also been reported in olive flounder (Paralichthys olivaceus) (31) and grouper (Epinephelus coioides) (32) following probiotic supplementation. Our findings in African catfish support the hypothesis that dietary inclusion of a multistrain combination of L. mesenteroides and L. lactis results in higher final body weight, weight gain, and specific growth rate than in the control group. Similarly, the use of multiple probiotic strains—including L. mesenteroides—has been shown to increase growth performance in Javanese carp (Puntius gonionotus) (19). In the present study, fish receiving the probiotic-supplemented diet also exhibited improvements in feed intake and nutrient utilization. These results suggest that probiotic supplementation may increase feed palatability, thereby stimulating appetite and promoting growth.

Previous studies in Nile tilapia have demonstrated that probiotics can enhance growth and feed conversion by stimulating digestive enzyme activity and increasing nutrient bioavailability (33).

Probiotic colonization of the intestinal tract is known to promote microbial balance, improve gut morphology, and facilitate nutrient absorption (34–36). In our study, the enhanced growth performance and feed utilization observed in the probiotic group may be attributable to improved digestive function mediated by increased enzymatic activity. Supplementation with Lactobacillus species has been shown to increase feed digestibility and nutrient extraction (7). Digestive enzyme activity is closely linked to the digestive capacity of fish and can be rapidly affected by dietary composition (37–39).

The introduction of beneficial microbial strains may also contribute to protease production and enhanced protein hydrolysis into amino acids, thereby improving the nutritional status of fish. Furthermore, bacterial enzymatic activity can increase the availability of lipids, proteins, and dry matter (40), ultimately supporting the improved growth and nutrient utilization observed in this study.

The results of this study demonstrated an increase in both total bacterial and LAB counts in the intestinal microbiota of fish in the probiotic-treated groups. This increase is likely a contributing factor to the improved growth performance and feed utilization observed in these groups. Similarly, a significant increase in total bacterial abundance has been reported in fish fed probiotic-supplemented diets (13). The dietary concentration of probiotic bacteria directly influences their colonization efficiency in the gastrointestinal tract. This colonization process is believed to support enhanced immunity, overall welfare, and growth in fish (41).

Therefore, the improvements in growth and nutrient assimilation observed in this study may be attributable to increased colonization and adherence of probiotic microorganisms introduced through dietary supplementation. Several studies have shown that probiotic bacteria colonizing the intestinal tract produce a variety of growth-promoting metabolites and nutrients (19, 42). Furthermore, recent publications have emphasized the critical interaction between probiotic strains and intestinal epithelial cells in regulating mucosal immunity. This interaction enhances immune responses by modulating both the physical and immunological barrier functions of the intestinal lining (36, 43–46).

Our results also demonstrated that the immune response of fish was significantly enhanced following dietary supplementation with a probiotic mixture containing L. mesenteroides and L. lactis. Specifically, increased activities of lysozyme and bactericidal components were observed in both serum and mucus, along with elevated activities of the alternative complement pathway, SOD, and peroxidase. Similar immunostimulatory effects have been reported in fish such as rainbow trout (Oncorhynchus mykiss) (47), Labeo rohita (48), and olive flounder (P. olivaceus) (27) following probiotic supplementation.

The use of bacterial strains with complementary functional properties has been shown to enhance host immunity and promote intestinal health under experimental conditions (49). Additionally, external factors such as probiotics, vitamins, and nutrients have been shown to influence the activity of the alternative complement pathway in fish (50, 51). In the present study, the immune parameter most strongly affected was alternative complement pathway activity, which was significantly elevated in the probiotic-treated group. This observation is consistent with previous findings in studies involving probiotic supplementation in fish (26, 32).

It is well known that bactericidal activity plays a crucial role in the host’s defense system against pathogenic microorganisms (7, 52). In our study, the highest levels of bactericidal activity in both serum and mucus were observed in fish fed a diet supplemented with a combination of L. mesenteroides and L. lactis. According to the literature, fish lysozyme activity is influenced by various probiotic bacterial species. For example, dietary supplementation with L. mesenteroides in brown trout (Salmo trutta) has been shown to positively affect lysozyme activity (45). Similarly, L. lactis-enriched diets modulated lysozyme activity in grouper (E. coioides) (32). Our findings are consistent with these reports, demonstrating that supplementation with a mixture of L. mesenteroides and L. lactis enhanced serum lysozyme activity in African catfish.

When L. mesenteroides and L. lactis were administered separately, both increased serum peroxidase levels; however, their combination did not lead to a significant change in peroxidase content. Similar findings have been reported in sea bream (Sparus aurata) (20).

SOD is a key enzyme involved in the primary antioxidant defense system and plays a critical role in converting highly reactive superoxide radicals into the less harmful hydrogen peroxide. In our study, SOD activity was significantly elevated in all experimental groups. Similar findings have been reported in L. rohita, where dietary supplementation with Bacillus subtilis in combination with Pseudomonas aeruginosa and Lactobacillus plantarum resulted in increased antioxidant enzyme activity (26).

The epidermal mucus and skin of fish serve as essential components of the nonspecific defense mechanisms, forming a physical barrier that protects the organism from environmental stressors and pollutants (53). In the present study, fish fed diets containing L. mesenteroides and L. lactis exhibited significantly increased mucus secretion compared with the control group. Furthermore, supplementation with these probiotic strains had a more pronounced effect on the innate immune system of African catfish. Compared with single-strain formulations, diets containing multiple probiotic strains appear to modulate the host immune response more effectively.

These findings support the conclusion that well-designed multispecies or multistrain probiotic preparations may exert greater health-promoting effects in the vertebrate gastrointestinal tract than single-strain probiotics (54, 55).

The effects of intestinal probiotics vary widely and depend on multiple factors, including microbial species, dosage, origin, and duration of administration. Therefore, selecting appropriate probiotic strains is essential for formulating effective combinations capable of exerting synergistic effects. In this study, the combination of L. mesenteroides and L. lactis demonstrated strong potential to enhance the growth performance and immune response of African catfish. Further research is recommended to optimize administration protocols and evaluate the practical applications of such probiotic formulations in aquaculture production systems.

## Data Availability

The datasets presented in this study can be found in online repositories. The names of the repository/repositories and accession number(s) can be found in the article/supplementary material.

## References

[1] Tan HY Chen SW Hu SY. Improvements in the growth performance, immunity, disease resistance, and gut microbiota by the probiotic Rummeliibacillus stabekisii in Nile tilapia (Oreochromis niloticus). Fish Shellfish Immunol. (2019) 92:265–75. doi: 10.1016/j.fsi.2019.06.027, PMID: 31202962

[2] Paritova A Nurgaliyev A Nurgaliyeva G Abekeshev N Abuova A Zakirova F. The dietary effects of two strain probiotics (Leuconostoc mesenteroides, Lactococcus lactis) on growth performance, immune response and gut microbiota in Nile tilapia (Oreochromis niloticus). PloS One. (2024) 19. doi: 10.1371/journal.pone.0312580, PMID: 39446799 PMC11500904

[3] Won S Hamidoghli A Choi W Park Y Jang WJ Kong IS. Effects of Bacillus subtilis WB60 and Lactococcus lactis on growth, immune responses, histology and gene expression in Nile tilapia, Oreochromis niloticus. Microorganisms. (2020) 8. doi: 10.3390/microorganisms8010067, PMID: 31906334 PMC7023347

[4] Paritova AY Balji YA Murzakayeva GK Aytkozhina BZ Zhanabayev AA Kuzembekova GB. Isolation of lactobacillus strains with probiotic activity to be used as a fish feed supplement. Int J Veterinary Sci. (2024) 14:527–34. doi: 10.47278/journal.ijvs/2024.268

[5] Defoirdt T Boon N Sorgeloos P Verstraete W Bossier P. Alternatives to antibiotics to controlbacterial infections: luminescent vibriosis in aquaculture as an example. Trends Biotechnol. (2007) 25:472–9. doi: 10.1016/j.tibtech.2007.08.001, PMID: 17719667

[6] Paritova A Sarsembayeva N Slyamova A Buralhiev B. An experimental study of the effect of natural zeolite of Chankanay deposits on fish-breeding and biological and hematological parameters of the body of fish. Global Veterinaria. (2013) 11:348–51. doi: 10.5829/idosi.gv.2013.11.3.1142

[7] Paritova AY Zwierzchowskii G Issimov AM Murzakayeva GK. Method for obtaining a symbiotic for fish from isolated strains of lactobacilli. (2025) Astana, KazPatent, Kazakhstan Patent №2024/0277.

[8] Beck BR Kim D Jeon J Lee S Kim HK Kim O. The effects of combined dietary probiotics Lactococcus lactis BFE920 and Lactobacillus plantarum FGL0001 on innate immunity and disease resistance in olive flounder (Paralichthys olivaceus). Fish Shellfish Immunol. (2014) 42:177–83. doi: 10.1016/j.fsi.2014.10.035, PMID: 25449382

[9] Cavalcante RB Telli GS Tachibana L Dias DDC Oshiro E Natori MM. Probiotics, Prebiotics and Synbiotics for Nile tilapia: Growth performance and protection against Aeromonas hydrophila infection. Aquaculture Rep. (2020) 17:100343. doi: 10.1016/j.aqrep.2020.100343

[10] Jasim SA Hafsan H Saleem HD Kandeel M Khudhair F Yasin G. The synergistic effects of the probiotic (Lactobacillus fermentum) and cinnamon, Cinnamomum sp. powder on growth performance, intestinal microbiota, immunity, antioxidant defence and resistance to Yersinia ruckeri infection in the rainbow trout (Oncorhynchus mykiss) under high rearing density. Aquaculture Res. (2022) 53:5957–70. doi: 10.1111/are.16064

[11] Dawood MAO Koshio S. Recent advances in the role of probiotics and prebiotics in carp aquaculture: A review. Aquaculture. (2016) 454:243–51. doi: 10.1016/j.aquaculture.2015.12.033

[12] Dawood MAO Koshio S Ishikawa M El-Sabagh M Esteban MA Zaineldin AI. Probiotics as an environment-friendly approach to enhance red sea bream, Pagrus major growth, immune response and oxidative status. Fish Shellfish. Immunol. (2016) 57:170–8. doi: 10.1016/j.fsi.2016.08.038, PMID: 27542618

[13] Zorriehzahra MJ Delshad ST Adel M Tiwari R Karthik K Dhama K. Probiotics as beneficial microbes in aquaculture: An update on their multiple modes of action: A review. Vet Q. (2016) 36:228–41. doi: 10.1080/01652176.2016.1172132, PMID: 27075688

[14] Dawood MAO Zommara M Eweedah NM Helal AI. The evaluation of growth performance, blood health, oxidative status and immune-related gene expression in Nile tilapia (Oreochromis niloticus) fed dietary nanoselenium spheres produced by lactic acid bacteria. Aquaculture. (2020) 515:734571. doi: 10.1016/j.aquaculture.2019.734571

[15] Merrifield DL Balcázar JL Daniels C Zhou Z Carnevali O Sun YZ. Indigenous lactic acid bacteria in fish and crustaceans. In: Aquaculture nutrition: gut health, probiotics and prebiotics (Hoboken, New Jersey: John Wiley & Sons, Ltd) (2014). p. 128–68.

[16] Dawood MA Koshio S Ishikawa M Yokoyama S El Basuini MF Hossain MS. Effects of dietary supplementation of Lactobacillus rhamnosus or/and Lactococcus lactis on the growth, gut microbiota and immune responses of red sea bream, Pagrus major. Fish Shellfish Immunol. (2016) 49:275–85. doi: 10.1016/j.fsi.2015.12.047, PMID: 26766177

[17] Sohel AM Shahjahan M Hossain MK Sumi KR Hossain MS Abdul Kari Z. Effects of multispecies probiotics on growth, hematology, and gut health of stinging catfish (Heteropneustes fossilis) in biofloc system. Water. (2023) 15:2519. doi: 10.3390/w15142519

[18] Ramos MA Batista S Pires MA Silva AP Pereira LF Saavedra MJ. Dietary probiotic supplementation improves growth and the intestinal morphology of Nile tilapia. Animal. (2017) 11:1259–69. doi: 10.1017/S1751731116002792, PMID: 28077192

[19] Allameh SK Yusoff FM Ringø E Daud HM Saad CR Ideris A. Effects of dietary mono- and multiprobiotic strains on growth performance, gut bacteria and body composition of Javanese carp (Puntius gonionotus, Bleeker 1850). Aquaculture Nutr. (2016) 22:367–73. doi: 10.1111/anu.12265

[20] Yano T. Assay of hemolytic complement activity. In: Techniques in fish immunology (NJ: SOS Publications, Fair Haven) (1992). p. 131–41.

[21] Salinas I Abelli L Bertoni F Picchietti S Roque A Furones D. Monospecies and multispecies probiotic formulations produce different systemic and local immunostimulatory effects in the gilthead seabream (Sparus aurata L.). Fish shellfish Immunol. (2008) 25:114–23. doi: 10.1016/j.fsi.2008.03.011, PMID: 18442923

[22] Sun YZ Yang HL Ma RL Lin WY. Probiotic applications of two dominant gut Bacillus strains with antagonistic activity improved the growth performance and immune responses of grouper Epinephelus coioides. Fish shellfish Immunol. (2010) 29:803–9. doi: 10.1016/j.fsi.2010.07.018, PMID: 20637875

[23] Sun Y He M Cao Z Xie Z Liu C Wang S. Effects of dietary administration of Lactococcus lactis HNL12 on growth, innate immune response, and disease resistance of humpback grouper (Cromileptes altivelis). Fish shellfish Immunol. (2018) 82:296–303. doi: 10.1016/j.fsi.2018.08.039, PMID: 30125700

[24] Al-Dohail MA Hashim R Aliyu-Paiko M. Effects of the probiotic, Lactobacillus acidophilus, on the growth performance, haematology parameters and immunoglobulin concentration in African Catfish (Clarias gariepinus, Burchell 1822) fingerling. Aquaculture Res. (2009) 40:1642–52. doi: 10.1111/j.1365-2109.2009.02265.x

[25] Hooshyar Y Abedian Kenari A Paknejad H Gandomi H. Effects of Lactobacillus rhamnosus ATCC 7469 on Different Parameters Related to Health Status of Rainbow Trout (Oncorhynchus mykiss) and the Protection Against Yersinia ruckeri. Probiotics Antimicrob Proteins. (2020) 12:1370–84. doi: 10.1007/s12602-020-09645-8, PMID: 32246325

[26] Adeshina I Abubakar MI Ajala BE. Dietary supplementation with Lactobacillus acidophilus enhanced the growth, gut morphometry, antioxidant capacity, and the immune response in juveniles of the common carp, Cyprinus carpio. Fish Physiol Biochem. (2020) 46:1375–85. doi: 10.1007/s10695-020-00796-7, PMID: 32232615

[27] Beck BR Kim D Jeon J Lee SM Kim HK Kim OJ. The effects of combined dietary probiotics Lactococcus lactis BFE920 and Lactobacillus plantarum FGL0001 on innate immunity and disease resistance in olive flounder (Paralichthys olivaceus). Fish Shellfish Immunol. (2015) 42:177–83. doi: 10.1016/j.fsi.2014.10.035, PMID: 25449382

[28] Zhang H Wang H Hu K Jiao L Zhao M Yang X. Effect of dietary supplementation of lactobacillus casei YYL3 and L. Plantarum YYL5 on growth, immune response and intestinal microbiota in channel catfish. Anim (Basel). (2019) 9:1005. doi: 10.3390/ani9121005, PMID: 31757039 PMC6941169

[29] Xia Y Lu M Chen G Cao J Gao F Wang M. Effects of dietary Lactobacillus rhamnosus JCM1136 and Lactococcus lactis subsp. lactis JCM5805 on the growth, intestinal microbiota, morphology, immune response and disease resistance of juvenile Nile tilapia, Oreochromis niloticus. Fish Shellfish Immunol. (2018) 76:368–79. doi: 10.1016/j.fsi.2018.03.020, PMID: 29550602

[30] Suzer C Çoban D Kamaci HO Saka Ş. Firat K Otgucuoğlu Ö.. Lactobacillus spp. bacteria as probiotics in gilthead sea bream (Sparus aurata, L.) larvae: Effects on growth performance and digestive enzyme activities. Aquaculture. (2008) 280:140–5. doi: 10.1016/j.aquaculture.2008.04.020

[31] Heo WS Kim YR Kim EY Bai SC Kong IS. Effects of dietary probiotic, Lactococcus lactis subsp. lactis I2, supplementation on the growth and immune response of olive flounder (Paralichthys olivaceus). Aquaculture. (2013) 376:20–4. doi: 10.1016/j.aquaculture.2012.11.009

[32] Sun YZ Yang HL Ma RL Song K Li JS. Effect of Lactococcus lactis and Enterococcus faecium on growth performance, digestive enzymes and immune response of grouper Epinephelus coioides. Aquaculture Nutr. (2012) 18:281–9. doi: 10.1111/j.1365-2095.2011.00894.x

[33] Xia Y Cao J Wang M Lu M Chen G Gao F. Effects of Lactococcus lactis subsp. lactis JCM5805 on colonization dynamics of gut microbiota and regulation of immunity in early ontogenetic stages of tilapia. Fish Shellfish Immunol. (2019) 86:53–63. doi: 10.1016/j.fsi.2018.11.022, PMID: 30428393

[34] Adeoye AA Yomla R Jaramillo-Torres A Rodiles A Merrifield DL Davies SJ. Combined effects of exogenous enzymes and probiotic on Nile tilapia (Oreochromis niloticus) growth, intestinal morphology and microbiome. Aquaculture. (2016) 463:61–70. doi: 10.1016/j.aquaculture.2016.05.028

[35] Iorizzo M Albanese G Letizia F Testa B Tremonte P Vergalito F. Probiotic potentiality from versatile Lactiplantibacillus plantarum strains as resource to enhance freshwater fish health. Microorganisms. (2022) 10:463. doi: 10.3390/microorganisms10020463, PMID: 35208917 PMC8877946

[36] Thomas CM Versalovic J. Probiotics-host communication: modulation of signaling pathways in the intestine. Gut Microbes. (2010) 1:148–63. doi: 10.4161/gmic.1.3.11712, PMID: 20672012 PMC2909492

[37] Wells JM. Immunomodulatory mechanisms of lactobacilli. Microbial Cell factories. (2011) 10:S17. doi: 10.1186/1475-2859-10-S1-S17, PMID: 21995674 PMC3231924

[38] Liu W Ren P He S Xu L Yang Y Gu Z. Comparison of adhesive gut bacteria composition, immunity, and disease resistance in juvenile hybrid tilapia fed two different Lactobacillus strains. Fish shellfish Immunol. (2013) 35:54–62. doi: 10.1016/j.fsi.2013.04.010, PMID: 23608032

[39] Balcázar JL De Blas I Ruiz-Zarzuela I Vendrell D Calvo AC Márquez I. Changes in intestinal microbiota and humoral immune response following probiotic administration in brown trout (Salmo trutta). Br J Nutr. (2007) 97:522–7. doi: 10.1017/S0007114507432986, PMID: 17313714

[40] Devi G Harikrishnan R Paray BA Al-Sadoon MK Hoseinifar SH Balasundaram C. Effect of symbiotic supplemented diet on innate-adaptive immune response, cytokine gene regulation and antioxidant property in Labeo rohita against Aeromonas hydrophila. Fish shellfish Immunol. (2019) 89:687–700. doi: 10.1016/j.fsi.2019.04.036, PMID: 31002929

[41] Slyamova A Sarsembayeva N Valdovska A Micinski J Ussenbayev A Paritova A. Effects of antibiotic growth promoters on biochemical and haematological parameters of broiler chickens’blood. Res Rural Dev. (2016) 1:131–6. doi: 10.1016/j.fsi.2012.05.004, PMID: 22634255

[42] Iorizzo M Albanese G Letizia F Testa B Tremonte P Vergalito F. Probiotic potentiality from versatile Lactobacillus plantarum strains as resource to enhance freshwater fish health. Microorganisms. (2022) 10:463. doi: 10.3390/microorganisms10020463, PMID: 35208917 PMC8877946

[43] Nurgaliyev B Kushmukhanov Z Kereyev AK Taubaev U Sengaliyev Y Bayantassova S. The efficacy of licorice root extract on meat amino acid, fatty acid, vitamin, and mineral composition and productivity of quail. Veterinary World. (2024) 17:1017. doi: 10.14202/vetworld., PMID: 38911091 PMC11188887

[44] Jakupov IT Yeszhanova GT Mamytbekova GK. Biological activity and pharmaco-therapeutic efficiency of Calligonum leucocladum B. dosage forms in the treatment of endometritis of cows. Adv Anim. Vet Sci. (2023) 11:1200–8. doi: 10.17582/journal.aavs/2023/11.7.1200.1208

[45] Moldagaliyeva D Sarsembaeva N Uzakov Y Lozovicka B. Probiotics in the creation of fish-based herodietic half-finished products. Slovak J Food Sci. (2024) 18:174–84. doi: 10.5219/1934

[46] Paritova A Biltebayevna N Kuzembekova G Valieva Z Sarybaeva D. The chemical composition and nutritional value of fish meat while using as a feed additive zeolite of Chankanay origin. Res Rural Dev. (2013) 1:163–7.

[47] Sarsembayeva NB Akkozova AS Abdigaliyeva TB Abzhalieva AB Aidarbekova AB. Effect of feed additive “Ceobalyk” on the biological and microbiological parameters of African sharptooth catfish (Clarias gariepinus). Veterinary world. (2021) 14:669. doi: 10.14202/vetworld.2021.669-677, PMID: 33935413 PMC8076447

[48] Moldagaliyeva DZ Sarsembayeva N Uzakov YM Biyashev B Salimgereyeva BZ Baitilessova D. Study on the biological drug enterocol’s effect on the nile tilapia breeding. Open Agric J. (2024) 18. doi: 10.2174/011874331531382224060311211

[49] Sarsembayeva NB Moldagaliyeva DZ Uzakov YM Abdigaliyeva TB Biltebay AN. Effects of new mixed feed based on nontraditional feed additives on fatty acid profile of Tilapia (Oreochromis Niloticus). IJRTE. (2019) 8:3790–4. doi: 10.35940/ijrte.A1362.078219

[50] Cerezuela R Guardiola FA González P Meseguer J Esteban MÁ. Effects of dietary Bacillus subtilis, Tetraselmis chuii, and Phaeodactylum tricornutum, singularly or in combination, on the immune response and disease resistance of sea bream (Sparus aurata L.). Fish Shellfish Immunol. (2012) 33:342–9. doi: 10.1016/j.fsi.2012.05.004, PMID: 22634255

[51] Mohammadian T Nasirpour M Tabandeh MR Mesbah M. Synbiotic effects of β-glucan, mannan oligosaccharide and Lactobacillus casei on growth performance, intestine enzymes activities, immune-hematological parameters and immune-related gene expression in common carp, Cyprinus carpio: An experimental infection with Aeromonas hydrophila. Aquaculture. (2019) 511:634197. doi: 10.1016/j.aquaculture.2019.06.011

[52] Irianto A Austin B. Probiotics in aquaculture. J Fish Dis. (2002) 25:633–42. doi: 10.1046/j.1365-2761.2002.00422.x

[53] Hernandez LHH Barrera TC Mejia JC Mejia GC Del Carmen M Dosta M. Effects of the commercial probiotic Lactobacillus casei on the growth, protein content of skin mucus and stress resistance of juveniles of the Porthole livebearer Poecilopsis gracilis (Poecilidae). Aquaculture Nutr. (2010) 16:407–11. doi: 10.1111/j.1365-2095.2009.00679.x

[54] Timmerman HM Koning CJ Mulder L Rombouts FM Beynen AC. Monostrain, multistrain and multispecies probiotics—A comparison of functionality and efficacy. Int J Food Microbiol. (2004) 96:219–33. doi: 10.1016/j.ijfoodmicro.2004.05.012, PMID: 15454313

[55] Wang Y Li A Jiang X Zhang H Mehmood K Zhang L. Probiotic potential of leuconostoc pseudomesenteroides and lactobacillus strains isolated from yaks. Front Microbiol. (2018) 9. doi: 10.3389/fmicb.2018.02987, PMID: 30564222 PMC6289064

